# The irony of Parkinson's disease: Converging mechanisms of redox imbalance and ferroptosis

**DOI:** 10.1016/j.redox.2026.104239

**Published:** 2026-05-27

**Authors:** Lauren E. Cooke, Emily Rocha, Roberto DiMaio, Adam C. Straub, Marco Fazzari

**Affiliations:** a Department of Pharmacology and Chemical Biology, University of Pittsburgh, Pittsburgh, PA, USA; b Pittsburgh Institute for Neurodegenerative Disease, University of Pittsburgh, Pittsburgh, PA, USA; c Heart, Lung, Blood and Vascular Medicine Institute, University of Pittsburgh, Pittsburgh, PA, USA; d Center for Microvascular Research, University of Pittsburgh, Pittsburgh, PA, USA; e Department of Neurology, University of Pittsburgh, Pittsburgh, PA, USA

**Keywords:** Ferroptosis, Parkinson's disease, Oxidative stress, Selective vulnerability, Neuropharmacology

## Abstract

Parkinson's disease (PD) is the second most common neurodegenerative disease worldwide and its prevalence will increase with population aging. PD is characterized by progressive degeneration of dopaminergic neurons in the substantia nigra pars compacta (SNpc), leading to severe motor and debilitating non-motor symptoms. Current therapies provide symptomatic relief without preventing the progressive nigrostriatal neurodegeneration. Unfortunately, clinical trials investigating single-target drugs and antioxidant supplementation have not provided robust clinical responses. Since PD is a multifactorial disease involving mitochondrial dysfunction, oxidative stress, α-synuclein aggregation, and neuroinflammation, the classical “one-drug-one-target” philosophy may be ineffective in preventing progression of the disease, while “one-drug-multiple-targets” approaches may offer greater neuroprotection. This review summarizes PD-related pathogenic events and potential disease-modifying strategies, with a particular focus on ferroptosis, a regulated iron-dependent cell death mechanism that has recently emerged as a key driver of dopaminergic degeneration. By synthesizing recent iron chelators- and antioxidant-based clinical trials, repurposed drugs and emerging preclinical pleiotropic strategies, we advocate for an integrated, multi-targeted approach to effectively halt the progression of PD.

## Highlights

• Parkinson's disease is a multifactorial disease, involving converging pathogenic mechanisms.
• Ferroptosis influences multiple pathogenic mechanisms of PD.
• An effective therapeutic must target multiple pathogenic mechanisms of PD.

## Graphical abstract

**Figure:**
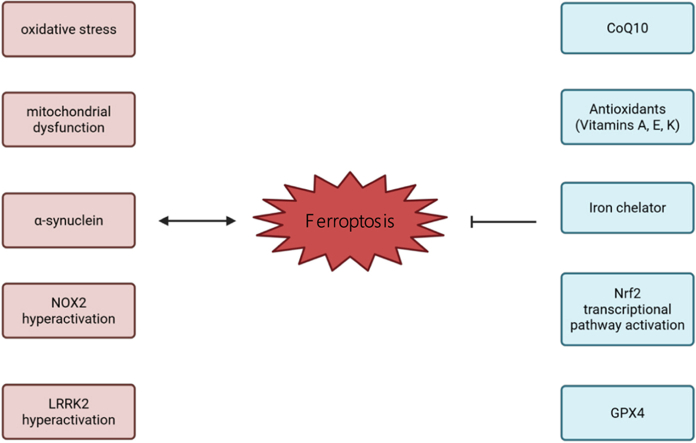

## 1 Introduction to Parkinson's disease

Parkinson's disease (PD) is the second most common neurodegenerative disease worldwide, with its incidence projected to rise significantly alongside an aging global population [1,2]. Within the United States alone, approximately 90,000 people are diagnosed with PD annually, contributing to an estimated worldwide prevalence of more than 10 million cases [3]. While the vast majority of PD cases are idiopathic [4,5], PD is likely driven by a complex interplay of environmental exposure and genetic predispositions [6,7]. Some environmental risk factors include exposure to pesticides, such as rotenone and paraquat [8], as well as industrial solvents like perchloroethylene and trichloroethylene [6]. These toxicants interact with both common risk variants and highly penetrant genetic mutations, such as *LRRK2, SNCA, PARKIN, PINK1, DJ1, CHCHD2,* and *VPS35* [5,7,9], to serve as critical drivers of the underlying pathophysiology.

PD is characterized by the progressive degeneration of a subset of dopaminergic neurons projecting from the Substantia Nigra pars compacta (SNpc) to the dorsal striatum [[10], [11], [12]]. This neurodegenerative process reduces striatal dopamine availability and disrupts the regulatory basal ganglia motor circuits, leading to the cardinal motor symptoms of PD [13,14]: tremors at rest, rigidity, bradykinesia (slowness of movement), and postural instability, often accompanied by hunched posture and freezing of gait [15]. Notably, these motor symptoms are typically preceded by up to two decades of prodromal non-motor symptoms, such as constipation, neuropsychiatric manifestations (anxiety, depression, or hallucinations), hyposmia, and REM sleep disorder [[15], [16], [17], [18], [19], [20], [21]].

## 2 Current therapeutic management and challenges

Levodopa is the undisputed “gold standard” treatment for PD because of its ability to cross the blood brain barrier (BBB) and restore dopamine levels within the central nervous system. [22]. To optimize central bioavailability, it is standard practice to co-administer levodopa with carbidopa, an aromatic l-amino acid decarboxylase inhibitor, which prevents peripheral metabolism. This synergistic combination increases the concentration of levodopa reaching the striatum but also significantly attenuates peripheral dopaminergic side effects like orthostatic hypotension [23]. Despite L-DOPAs effectiveness, it does not treat the disease – it merely alleviates the symptoms. Moreover, long-term levodopa therapy is often associated with motor complications such as dyskinesia and “on-off” motor fluctuations [23]. To address these limitations or provide adjunctive support, other strategies to help alleviate patient discomfort and provide additional symptomatic relief include: dopamine agonists like pramipexole and ropinirole, monoamine oxidase B (MAO-B) inhibitors (selegiline, rasagiline) and catechol-*O*-methyl transferase (COMT) inhibitors (entacapone, nitecapone) to inhibit metabolic degradation of levodopa and dopamine [23,24]. Anticholinergic drugs (trihexyphenidyl and benztropine) are mainly used to manage tremor, and adenosine A2A receptor antagonists may be used in combination with levodopa to reduce “off” periods [24,25]. While these treatments effectively alleviate motor symptoms, they do not offer relief for the swath of non-motor symptoms associated with PD. Consequently, patients often require a complex regimen of adjunctive medications, including benzodiazepines or selective serotonin reuptake inhibitors (SSRIs) for mood-related disorders and lubiprostone to manage autonomic dysfunction such as constipation [26]. Additional drugs are required for the management of cognitive decline, psychosis, and sleep disorders [23,25]. Collectively, while these current treatments provide vital symptomatic relief, they fail to halt or delay the progressive neurodegeneration, and are associated with significant side effects, underscoring the need for novel disease-modifying strategies.

## 3 Pathogenic mechanisms of Parkinson's disease

**Fig. 1:**
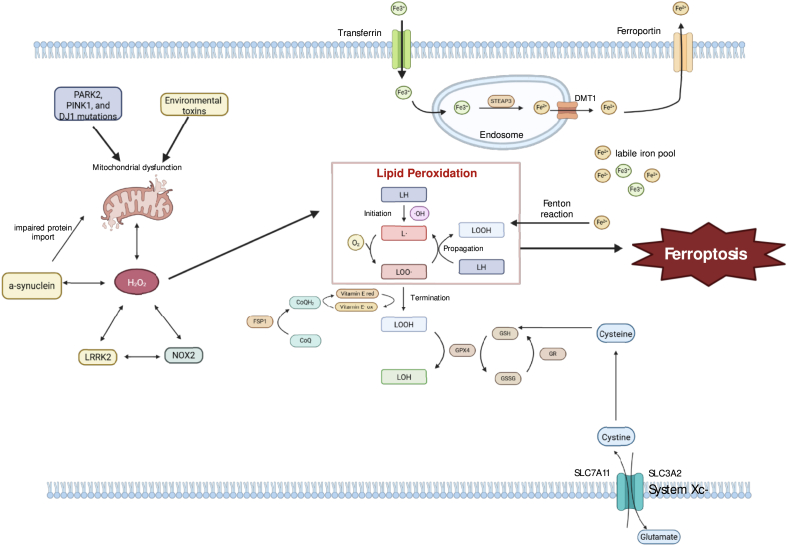
Interconnected pathogenic and ferroptotic mechanisms driving lipid peroxidation in Parkinson's disease. Schematic representation of the major molecular pathways contributing to dopaminergic neurodegeneration in PD. Mitochondrial dysfunction, α-synuclein aggregation, neuroinflammation, and iron accumulation exacerbate oxidative stress and promote lipid peroxidation. In parallel, ferroptosis-related processes—characterized by iron dysregulation, glutathione depletion, and impaired GPX4 activity—amplify redox imbalance and membrane lipid oxidation. Conversely, antioxidant systems and ferroptosis inhibitors (e.g., GPX4, NRF2, and iron chelators) mitigate lipid peroxidation and protect against ferroptotic cell death. The resulting feedback loop between PD pathology, lipid peroxidation, and ferroptosis establishes a self-propagating cycle of oxidative neuronal injury.

Because PD stems from a complex intersection of mitochondrial dysfunction, oxidative stress, α-synuclein aggregation, and neuroinflammation, the traditional “one-drug one-target” approach is increasingly viewed as insufficient to halt disease progression. In contrast, multi-target pharmacological strategies may provide broader neuroprotection by simultaneously targeting multiple pathogenic events. Known pathogenic events triggered by both genetic mutations and environmental toxicant exposures, ultimately converge to disrupt iron homeostasis inducing ferroptosis, a distinct cell death pathway that has emerged as a central mediator of neurodegeneration in PD (Fig. 1).

Nigrostriatal dopaminergic (DA) neurons exhibit autonomous pacemaking activity that depends on continuous Ca^2+^ influx, requiring tight regulation of CaMKIIα–Ca^2+^/calmodulin signaling and efficient mitochondrial Ca^2+^ buffering. While mitochondria help control cytosolic Ca^2+^, this process is coupled to reactive oxygen species (ROS) generation. As a result, these neurons maintain inherently elevated basal levels of Ca^2+^ and ROS, imposing a chronic metabolic and oxidative burden. This limited buffering capacity reduces their ability to cope with additional stress, helping explain why systemic exposure to mitochondrial toxicants, such as pesticides and organic solvents, leads to selective nigrostriatal damage [27,28].

### 3.1 Ferroptosis

Distinct from apoptosis, ferroptosis is a form of regulated cell death driven by iron-mediated accumulation of lipid peroxides [[29], [30], [31]]. Dysregulation of iron metabolism leads to an excess of labile iron (Fe^2+^) levels which catalyze the Fenton reaction with hydrogen peroxide to generate highly reactive hydroxyl radicals. Under healthy conditions, GSH scavenges ROS and helps maintain intracellular redox homeostasis, while low levels of ROS participate in normal redox signaling. When ROS production overwhelms the antioxidant buffering capacity, oxidative stress can drive lipid peroxidation. Excess reactive oxygen species (ROS) promote an unchecked attack on polyunsaturated fatty acids (PUFAs) within membrane phospholipids [29], producing lipid peroxyl radicals that propagate a self-amplifying chain reaction, progressively compromising membrane integrity. GPX4 is the cell's main antioxidant, using GSH to reduce phospholipid hydroperoxides into lipid alcohol. As lipid hydroperoxides accumulate in membranes, GSH levels deplete and the cell's antioxidant defenses such as glutathione peroxidase 4 (GPX4) or Vitamin E become overwhelmed, cells undergo membrane rupture and organelle dysfunction, culminating in non-apoptotic cell death characteristic of ferroptosis. The exacerbation of lipid peroxide accumulation is further driven by concurrent impairment of cellular detoxification systems, including the glutathione (GSH)-GPX4 axis and the cysteine-glutamate antiporter (System X_c_^−^) [31,32], as well as altered expression of ferroptosis-related genes (*SLC7A11, ACSL4, FTH1, NCOA4,* and *PLA2G6*) [33].

Postmortem analysis of PD brain tissue and experimental models of the disease recapitulate this scenario strongly, supporting the hypothesis that ferroptosis as a key pathogenic event in PD. Transferrin receptor 2 is significantly upregulated in surviving SNc DA neurons in post-mortem PD brains, suggesting that these neurons contain more iron in comparison to age matched control tissue. Moreover, transcriptomic data indicates decreased transferrin and total ferritin levels suggesting dysfunctional iron handling [34,35]. Transferrin normally sequesters iron in a safer, bioavailable form, limiting iron-driven oxidative damage. Therefore, a decrease in ferritin levels increases the potential for iron dysfunction and ferroptosis [36]. Furthermore, PD models show depleted glutathione (GSH) and GPX4 levels [33,37], alongside increased markers of lipid peroxidation, such as 4-HNE and malondialdehyde (MDA), underscoring ferroptosis as a hallmark of the disease's progression [33,38,39].

### 3.2 Endogenous and pharmacological protection against ferroptosis

The endogenous cellular mechanisms that mitigate ferroptosis and the propagation of lipid peroxidation have emerged as novel therapeutic targets to treat PD. These anti-ferroptotic strategies are thought to be disease modifying, rather than symptomatic relief (Table 1, Table 2).

**Table 1:** Preclinical therapeutic agents inhibiting ferroptosis.

Target/ Hypothesis	Therapeutic agent	Mechanism of action	Cellular Effects	In-vivo effects	Behavioral Effects	Reference
Antioxidant	Alpha lipoic acid (ALA)	Enzyme cofactor with iron chelator and antioxidant properties	PC12 cells with 6-OHDA-induced damage: ↑ GSH ↓ lipid peroxidation ↓ MDA ↓ Fe2^+^ × mitochondrial abnormalities (shrinkage and mitochondrial ridge)	MPTP-treated mice: ↑ TH-positive neurons and TH expression in the SN ↓ iron-staining cells in the SN ↓ total iron ↓ DMT1 expression ↑ FPN expression ↑ FTH1 expression ↑ xCT expression ↑ GPX4 expression ↑SIRT1 protein expression ↑ NRF2 protein expression	MPTP-treated mice: ↑ total distance and average speed during open field test ↑ pole-climbing ability ↓ immobility time during swim test	[144]
	Morroniside	Exerts neuroprotective, antioxidant, and anti-inflammatory effects, regulates oxidative stress	MPP + -treated PC12 cells: ↓ cell death caused by MPP^+^ and the Nrf2 inhibitor ML385 ↑ GSH ↑ mRNA levels of GPX4 and SLC7A11 ↑ Nrf2 and HO-1 × cellular damage, specifically in the mitochondria	MPTP-treated mice: × abnormalities in dopaminergic neuron morphology × mitochondrial morphology abnormalities ↑ tissue and organelle damage repair ↑ recovery of TH levels in the SN ↑ HO-1, GPX4, SLC7A11, FTH-1, and FPN	MPTP-treated mice: ↑ performance in the open-field and pole-climbing tests (only in the medium- and high-dose groups)	[86]
	Idebenone	Analog of CoQ10	None	Rats received rotenone*via*injection into the substantia nigra pars compacta and ventral tegmental area, Idebenone was administered through oral gavage: partially rescued markers of PD and ferroptosis.	Rotenone-treated rats: partially rescued motor impairment caused by rotenone.	[145]
	Resveratrol	Antioxidant and iron chelator. Activates SIRT1/Nrf2 pathway, SIRT3/FoxO3a pathway, and increases GPX4 and HO-1	Rotenone-induced microglial BV-2 cells: ↓ microglial activation and M1 polarization ↓ free iron, ROS, and MDA ↑ GSH ↑ Nrf2 and SLC7A11, and inhibited ↓ STAT1 and Keap1.	None	None	[146]
	Quercetin	Natural flavonoid, Nrf2-dependent mechanism of protection	M17 erastin or RSL3: × cell death × abnormal mitochondrial morphology ↓ lipid ROS ↑ GPX4 protein expression ↑ Nrf2 protein expression ↑ protein levels of GPX4, FTH, and SLC7A11. PC12 cells treated with MPP+: × cell death × abnormal mitochondrial structural changes ↑ Nrf2	MPTP-treated mice: × loss of TH-positive neurons in the SNpc and striatum ↑ Nrf2 expression levels ↑ GPX4 and SLC7A11 ↓ MDA levels and lipid peroxidation in the SNpc ↑ GSH ↑ SOD	MPTP-treated mice: ↑ rotarod performance	[147]
	Lapatinib	Antioxidant and Nrf2 promoter	None	Chronic rotenone model in rats: × loss of TH expression in the SN × loss of striatal dopamine levels ↓ 4-HNE, TFR1, and iron in the striatum ↑ Nrf2 expression, ↑GPX4 mRNA expression ↑ GSH ↓ ACSL4 expression.	Chronic rotenone model in rats: ↑rotarod performance ↑ grip-strength performance	[87]
	Curcumin	Radical scavenging, iron chelating, and anti-inflammatory properties	None	Rats with 6-OHDA lesioning: ↑ striatal dopamine ↑ Th positive neurons in the SN ↓ iron positive cells in the SN	None	[148]
	Baicalein	12/15-lipoxygenase inhibitor blocks PUFA peroxidation	Primary neurons treated with MPP+: No protective effect on MPP + -induced cell death. Primary astrocytes treated with MPP+: ↓ COX2 expression nuclear translocation of p65	MPTP-treated mice: × loss of TH-positive neurons in the SN × loss of TH-positive fibers in the striatum × astrocyte activation in the SN	MPTP-treated mice: ↑ rotarod performance	[149]
			SN4741 cells transfected with either W-syn gene or A53T gene: ↓ monomeric and aggregated α -syn ↑ cell viability in cells transfected with α -syn ↑ ratio of LC3B II/LC3B I	None	None	[150]
	Ferrostatin-1	Scavenges lipid peroxyl radicals in membranes, prevents iron-dependent lipid peroxidation	None	LPS-treated mice: ↓number of Iba-positive cells ↓ IL-6 and TNF-a levels in the hippocampus ↓ MDA and LPO ↑ SOD and GSH × mitochondrial structural abnormalities × iron deposits ↑ xCT, GPX4, TF, FTH expression	LPS-treated mice: × deficits in spatial-dependent memory × deficits in contextual-dependent memory × object recognition memory	[151]
	Liproxstatin-1	Blocks phospholipid peroxidation	NGF-PC12 cells treated with MPP+: ↑ DMT1, TfR1, GPX4, and FTH1 gene and protein expression ↓ ACSL5 expression	None	None	[152]
System Xc^—^GSH-GPX4 axis	Thioredoxin-1 upregulation	Antioxidant that supports GPX4 activity.	PC12 cells treated with MPP + overexpressing Trx-1: × decrease in cell viability ↑ LDH ↑ GSH ↑ GSH/GSSG ratio	Transgenic h-Trx-1 MPTP-treated mice: × loss of TH-positive neurons in the SNpc ↑ GPX4 levels	Transgenic h-Trx-1 MPTP-treated mice: ↑ grip-strength performance ↑ rotarod performance	[153]
Autophagy inducer	Rapamycin	Inhibits mTORC1	MPP + -treated PC12 cells: × decrease in cell viability ↑ LC3II/LC3I ↓ P62	MPTP-treated mice: ↓ α-synuclein expression × decrease of TH-positive neurons ↑ SLC7A11 and GPX4 expression	MPTP-treated mice: × weight loss	[154]
Iron chelation	PBT434 (8-hydroxyquinazolin-4(3H)-one)	Binds iron to abolish pathological reaction with α-synuclein, but with an affinity that is designed not to disrupt physiological iron homeostasis	None	MPTP-treated mice: ↓ 8-isoprostane in the SNpc ↓ α-syn ↓ ferroportin in the SNpc hA53T mouse model: ↓ nigral insoluble α-syn ↑ nigral ferropotin Dogs and rats undergoing 28-day dose-tolerability study: ↓ α-syn levels	None	[155]
	Deferoxamine		NGF-PC12 cells with FAC-induced iron overload: ↓ ROS ↓ mitochondrial membrane potential ↓ ACSL4 expression ↑ GPX4 and FTH1 expression MPP ^+^ treated NGF-PC12 cells: ↓ ROS ↓ intracellular oxidative stress ↑ cell viability ↑ mitochondrial membrane potential ↓ DMT1, ACSL4, and TFR11 expression ↑ GPX4 and FTH1 expression	None	None	[152]
	VK-28 (5-[4-(2-hydroxyethyl) piperazine-1-ylmethyl]-quinoline-8-ol)	Iron chelator and MAO inhibitor	None	Sprague-Dawley Rats treated with 6-OHDA: × Th-positive neuronal loss in the SN × change in striatal dopamine metabolites the striatum	Sprague-Dawley Rats treated with 6-OHDA: × weight loss	[156]
	M-30 (5-(N-methyl-N-propargyaminomethyl)-8-hydroxyquinoline)	Iron chelator	None	Mice injected with lactacystin: × loss of TH-positive neurons in the SN × change in striatal dopamine metabolites the striatum ↓ iron accumulation in the ventral midbrain ↓ microglial activation	Mice injected with lactacystin: ↑ distance traveled ↑ movement time ↑ Rotarod performance	[157]
	Apoferritin	Iron-free form of ferritin	None	MPTP-treated mice: × loss of TH-positive neurons in the SN ↓ Iba-1-positive microglia ↓ iron-positive cells in the SN ↓ ACSL4 expression ↑ FSP1 expression	MPTP-treated mice: × weight loss ↑ Rotarod performance ↑ pole-climbing performance ↑ open-field test performance	[158]
Nrf2 activator	Dimethyl fumarate (DMF)		MND6 cells: some modulation of p62-dependent autophagy	Nrf2 positive and KO mice injected with rAAV6 viral vector to express human α-synuclein: In the Nrf2 positive mice: ↑ Nrf2 protein and its downstream antioxidative genes × loss of TH-positive neurons in the SN	Nrf2 positive and KO mice injected with rAAV6 viral vector to express human α-synuclein: In the Nrf2 positive mice: ↑performance in measured contralateral body swing in	[88]
Mitochondrial dysfunction	MitoQ ([10-(4,5-dimethoxy-2-methyl-3,6-dioxo-1,4-cyclohexadien-1-yl) decyl]triphenylphosphonium)	Accumulates within the mitochondria and is continually recycled to the active ubiquinol antioxidant by complex II of the respiratory chain.	SH-SY5Y cells treated with 6-OHDA: ↓ mitochondrial fission ↓ mitochondrial translocation of Drp1 and Bax	None	None	[116]
			MPP + -treated Primary cultures of rat MDNs: ↑ cellular dopamine levels. MPP + -treated N27 cells: ↑ TH expression ↑ mitochondrial membrane potential ↑ cell viability	MPTP-treated mice: × loss of TH-positive neurons in the SN × loss od dopamine and its metabolites	MPTP-treated mice: ↑ horizontal activity ↑ vertical activity ↑ total distance traveled ↑ total movement ↑ rearing time Rotarod performance	[117]
	Metformin		PC12 cells: ↑ GPX4, SLC7A11, IL-1B, and IL-6 expression ↓ cell damage ↓ abnormal mitochondrial morphology	Spinal cord injury Sprague Dawley rats: ↑ GPX4 protein levels ↓ MDA ↓ IL-1B and IL-6 ↑ Nrf2 pathway and NQO1	Spinal cord injury Sprague Dawley rats: ↑ hind limb motor function	[131]
			PC-12 cells treated with H_2_O_2_: ↑ cell viability ↓ LDH ↓ apoptosis ↓ ROS ↑ mitochondrial membrane potential ↑ AMPK phosphorylation Primary hippocampal neurons treated with H_2_O_2_: ↑ cell viability in AMPK-dependent manner	None	None	[133]
			PC-12 cells treated with H_2_O_2_: ↑ cell viability PC12 cells/SH-SY5Y cells/primary neurons exposed to cadmium: ↑ cell viability ↓ apoptosis (nuclear fragmentation and condensation) ↓ phosphorylation of JNK and downstream phosphorylation of c-Jun ↓ ROS-dependent PP5/AMPK-JNK signaling pathway	None	None	[132]
			None	MPTP-treated mice: ↓ oxidative stress measured by an elevation of SOD, CAT and GSH ↓ midbrain LPO ↑ BDNF in midbrain × loss of TH-positive neurons in the SNpc	MPTP-treated mice: ↑ rotarod performance ↑ open field test performance	[134]
Dopamine Receptor agonist	D-512	Potent D2/D3 receptor agonist	6-OHDA-treated MN9D cells: ↑ cell viability ↓ caspase-3 activity ↓ changes in nuclear morphology ↑ TH expression MPP + -treated MN9D cells: ↑ cell viability SNP-treated MN9D cells: ↓ lipid peroxidation	None	6-OHDA-treated mice: ↑rotational activity in lesioned mice	[159]
			6-OHDA-treated PC-12 cells: ↑ cell viability ↓ intracellular oxidative stress ↓ SNP-induced lipid peroxidation ↓ nuclear condensation ↓ DNA fragmentation	MPTP-treated mice: × loss of TH-positive neurons in the SNpc × loss of striatal dopamine levels	MPTP-treated mice: ↑ pole test performance	[142]

**Table 2:** Ferroptosis-inhibiting and PD-related therapies in clinical trials.

Target/ Hypothesis	Therapeutic agent	Mechanism of action	Modality	Phase of study/Status	Outcome	Clinical Trial	References
Iron chelation	Deferiprone		Twice daily oral dose	Phase II- completed	Good safety profile and decreases iron in specific brain regions.	NCT01539837	[46]
				Phase II-completed	SN iron reduction and motor improvement in early-stage PD.	NCT00943748	[47]
				Phase II-completed	Brain iron reduction, but no significant clinical benefit on UPDRS scores	NCT02655315	[47,160] [48,161]
Antioxidant	CoQ10	Antioxidant protection and mitochondrial ETC support.	Chewable wafers	Phase II- completed	No change in UPDRS scores.	NCT00004731	[162]
			Oral capsule	Phase III- completed	No evidence of clinical benefit.	NCT00740714	[68]
			Oral tablets	Phase II/III- completed	Safe and well-tolerated, some improvement in UPDRS scores.	NCT00943748	[163]
	Tocovid Suprabio (HOV-12020) (Palm oil-derived vitamin E, tocotrienol)	Vitamin E terminates lipid peroxidation	Oral tablet	Phase II- ongoing	Ongoing study: assessing change in UPDRS I and II scores.	NCT04491383	
	Omega-3 + vitamin E		1000 mg omega-3 (flaxseed oil) + 400 IU vitamin E		Modest improvements in UPDRS and some metabolic markers (hs-CRP, GSH), interpreted as anti-inflammatory/antioxidant effects; not powered or designed as disease-modifying	No NCT number listed in the publication; conducted as a small single-center RCT without registry entry	[79]
	Deprenyl and tocopherol		Vitamin E (α-tocopherol) 2000 IU/day orally, with or without selegiline, in early PD	Study completed	Vitamin E did not delay disability or levodopa start	No NCT number (ran before ClinicalTrials.gov registration was standard)	[78]
Inhibiting LRRK2 kinase activity	BIIB122 (DNL151	Binds the ATP-binding pocket of the LRRK2 kinase domain, blocking kinase activity.	Daily oral dose	Phase IIa- ongoing	Assessing safety and biomarkers.	NCT06602193	[164,165]
				Phase 2b- Recruiting	Assessing MDS-PDRS progression.	NCT05348785	[103]
	NEU-411	Selective kinase inhibitor	Oral tablet/capsule	Phase 2- recruiting	Measuring safety and efficacy.	NCT06680830	[166]
Preventing α-syn aggregation/accumulation	ACI-7104.056	Anti-α-synuclein active immunotherapy	Vaccine	Phase II-recruiting	Ongoing trial	NCT06015841	[167]
	AFFITOPE PD01A	Generates an immune response against the oligomeric forms of α-syn by using a modified eight-amino acid peptide to mimic the C-terminal amino acids of α-syn.	Vaccine	Phase I-completed	Safe and well-tolerated, showed target engagement, not powered to detect clinical efficacy.	NCT02618941	[125,[168], [169], [170]]
				Phase I-completed	Follow-up to assess safety and clinical activity. No other results posted.	NCT02216188	
				Phase I-completed	Assessed safety and tolerability, no further results posted.	NCT01568099	
				Phase I-completed	Saw a sustained α-synuclein-specific antibody response	NCT01885494	
				Phase I-withdrawn	None- study withdrawn	NCT02758730	
	AFFITOPE PD03A		Vaccine	Phase I-completed	Safe and well-tolerated	NCT02267434	[126]
	Buntanetap/Posiphen	Blocks the production of multiple harmful proteins (amyloid beta, tau, α-synuclein, TDP43) that contribute to neurodegeneration in PD and Alzheimer's disease.	Oral capsule	Phase III- ongoing	Improvements in the Movement Disorder Society-Unified Parkinson's Disease Rating Scale (MDS-UPDRS) Parts II (non-motor), III (motor), and Total scores.	NCT05357989	[171,172]
	Cinpanemab	Targets α-syn, binding to residues 1–10 of the N-terminal.	Antibody	Phase I-completed	Well-tolerated and good target engagement.	NCT02459886	[[173], [174], [175], [176], [177]]
				Phase I-terminated	Safe and well-tolerated. Demonstrated acceptable pharmacokinetics and target engagement.	NCT03716570	
				Phase II-terminated	Study terminated.	NCT03318523	
	Lu AF82422	Humanized monoclonal IgG1 antibody.	Antibody	Phase I-completed	Safe and well-tolerated. Good target engagement and approved pharmacokinetics.	NCT03611569	[178,179]
				Phase I-active	Ongoing, no results yet.	NCT06258720	
	MEDI1341	A high-affinity monoclonal antibody that targets a C-terminal epitope on both monomeric and aggregated forms of α-syn.	Antibody	Phase I-completed	Safe and well-tolerated, showed dose-dependent increases in pharmacokinetic analysis. Showed good target engagement.	NCT03272165	[180,181]
				Phase I-completed	Safe and well-tolerated. Produced dose-dependent suppression of CSF free α-synuclein.	NCT04449484	
	Prasinezumab	Humanized IgG1 monoclonal antibody targeting aggregated α-syn. Reduces a neurotoxic, truncated form of α-syn and prevents α-syn propagation between cells.	Antibody	Phase I-completed	Safe and well-tolerated, good target engagement, reduction in circulating free s-synuclein.	NCT02095171	[[127], [128], [129], [130],[182], [183], [184]]
				Phase I-completed	Confirmed safety and tolerability. Predictable pharmacokinetic data and good target engagement. No significant improvement on MDS-UPDRS.	NCT02157714	
				Phase II-active	No significant changes in MDS-UPDRS.	NCT03100149	
				Phase IIb-active	Ongoing trial- measuring changes in MDS-UPDRS scores	NCT04777331	
	Risvodetinib (IkT-148009)	c-Abl tyrosine kinase inhibitor.	Oral dose	Phase II- ongoing	Assessing safety, tolerability, and pharmacokinetics	NCT05424276	[185]
	UB-312	Target the insoluble α-syn protein by producing antibodies against the C-terminal epitope of α-syn 97–135.	Vaccine	Phase I-completed	Safe and well-tolerated. Measurable Anti- α-synuclein response in the serum.	NCT04075318	[186]
				Phase Ib-active	Ongoing- measuring changes in MDS-UPDRS scores.	NCT05634876	
	BIA 28-6156	Allosteric activator of glucocerebrosidase (GCase), improving lysosomal function and sphingolipid recycling, specifically treating GBA1-assocaited PD.	Oral tablet	Phase II- ongoing	Well-tolerated in phase Ib trials, currently assessing the efficacy, safety, tolerability, pharmacodynamics, and pharmacokinetics of 2 fixed dose levels of BIA 28-6156 (10 and 60 mg/day) in approximately 237 subjects with genetically confirmed GBA-PD.	NCT05819359	[187,188]
ERK/IL-1B inflammation	Bezisterim (NE3107)	Inhibits extracellular signal-regulated kinase (ERK) signaling via multiple nuclear factor kappa B subunit 1 (NFkB) stimulated pro-inflammatory signaling.	Oral capsule	Phase II- completed	Well tolerated and patients saw improvements in sleep, fatigue and restlessness and improvements in motor control.	NCT05083260	[189,190]
				Phase II- Ongoing	Measuring Change from baseline in the MDS-UPDRS Part I, II, and III in patients with early-stage PD.	NCT06757010	
Mitochondrial dysfunction	MitoQ	Accumulates within the mitochondria and is continually recycled to the active ubiquinol antioxidant by complex II of the respiratory chain.	Oral tablets	Phase II- completed	Safe and well tolerated. It did not slow progression of PD based on UPDRS. There was a potential for insufficient brain penetration described in the results paper.	NCT00329056	[118]
GCase activity	Amborxol	GCase chaperone	Oral administration	Phase II- completed	Safe and well-tolerated. Showed an increase of Ambroxol in the CSF, an increase of CSF GCase activity, and an improvement in UPDRS scores.	NCT02941822	[135,136]
				Phase 3- Ongoing	N/A	(NCT05778617)	N/A
GLP-1 agonists	Exenatide	GLP1-agonist	Subcutaneous injection	Phase II- completed	Improved UPDRS scores, augmented tyrosine phosphorylation of insulin receptor substrate 1, elevated expression of downstream substrates including Akt, and phosphorylated mechanistic target of rapamycin (mTOR)	(NCT01971242)	[140,141]
	Liraglutide	GLP1-agonist	Subcutaneous injection			NCT02953665	
			Subcutaneous injection			NCT01843075	

#### 3.2.1 Iron chelation

The maintenance of iron homeostasis serves as primary defense mechanism against ferroptosis. Within a cell, the cytosolic labile iron pool is coordinated to PCBP1 and PCBP2, ensuring iron is available for essential redox reactions [[40], [41], [42]]. Since ferroptosis is triggered by accumulation of labile Fe^2+^, that catalyzes the Fenton reaction and drives lipid peroxidation [40], cells employ sophisticated chelation strategies to sequester redox-active iron. Systemically, transferrin chelates iron in the circulation to prevent oxidative damage during transport and deliver of iron to tissues via transferrin receptors [43]. Intracellularly, ferritin stores mineralized iron, releasing it in a highly controlled manner to limit the labile iron pool and to reduce cellular susceptibility to ferroptosis [44,45].

Given the dependence of ferroptosis on the catalytic activity of Fe^2+^, pharmacological chelators have been investigated in multiple clinical trials for their potential to reduce the redox-active iron pool. The iron chelator deferiprone (DFP) yielded promising results in a pilot study of 22 patients with PD. Daily administration of 30 mg/kg DFP for 6 months led to a modest improvement in motor-Unified Parkinson's Disease Rating Scale (UPDRS) scores and a significant reduction in iron concentrations across several brain regions, but not the SNpc [46]. Similarly, a subsequent trial of 48 patients suggested that prolonged DFP treatment could improve motor scores compared to the placebo group. DFP also increased striatal dopamine levels as measured by HPLC and MRI-PRET [47]. However, these early indications of efficacy have not been supported by larger, more robust clinical trials. Most notably, a comprehensive study of 372 patients with early-stage levodopa-naive PD demonstrated that 36 weeks of DFP treatment (15 mg/kg/day) decreased nigrostriatal iron content but was associated with worse symptoms than placebo. [48]. Consequently, while iron chelation successfully modulated iron brain levels, it is insufficient as monotherapy to arrest or slow down the progression of PD.

#### 3.2.2 System X_c_^−^-GSH-GPX4 axis

The System X_c_^−^-GSH-GPX4 axis serves as a primary endogenous defense against ferroptosis by maintaining cellular antioxidant capacity. Central to this pathway is the cystine-glutamate antiporter system X_c_^−^, a heterodimer containing the light-chain subunit SLC7A11, which facilitates the uptake of extracellular cystine in exchange for intracellular glutamate. Once internalized, cystine is then reduced to cysteine, the rate-limiting precursor for the synthesis of GSH [31]. As a vital cofactor, GSH facilitates the catalytic cycle of glutathione peroxidase 4 (GPX4) [49], a selenoenzyme that specifically reduces lipid hydroperoxides into non-toxic alcohols, thereby preserving membrane integrity and arresting the ferroptotic cascade [30].

Evidence suggests that dysregulation of X_c_^−^-GSH-GPX4 axis plays a significant role in PD pathogenesis. Specifically, hypermethylation of the SLC7A11 promoter has been linked to its downregulation in PD, leading to diminished GSH synthesis and heightened vulnerability to oxidative stress [50]. Analysis of post-mortem PD brains consistently reveal elevated concentrations of iron and lipid peroxidation products as well as significantly reduced GSH levels [45,51,52] [37]. Beyond its role in GSH synthesis, SLC7A11 may further protect neurons by inhibiting the expression of ALOX12, an oxygenase that directly promotes lipid peroxidation [53].

Therapeutic strategies aimed at replenishing GSH levels via the orally bioavailable precursor and free-radical scavenger N-acetylcysteine (NAC) have shown promising results. In preclinical 6-OHDA and rotenone rodent models of PD, NAC administration improved dopamine transporter (DAT) levels, reduced dopaminergic neurodegeneration and alleviate motor deficits [[54], [55], [56]]. In clinical trials, NAC has successfully increased both systemic and brain GSH levels [57,58]. A 90-day pilot study in PD patients reported increased striatal DAT binding and corresponding improvements in UPDRS scores when NAC was used in combination with standard dopaminergic therapy [59].

Despite the potential of NAC, other GSH-delivery methods have faced challenges in demonstrating clinical efficacy. Although both intravenous and intranasal GSH administration to PD patients were well-tolerated in early clinical trials, neither resulted in significant changes of UPDRS [60] or measurable symptomatic improvement (NCT01398748) [61]. Consequently, a current Phase 1 trial is shifting toward alternative GSH precursors, such as gamma-glutamylcysteine supplementation (NCT07064005). While many GSH-targeted therapies are in the early clinical phases, the X_c_^−^-GSH-GPX4 axis remains a promising target for preventing ferroptosis in PD.

#### 3.2.3 Antioxidant strategies

##### 3.2.3.1 Coenzyme Q_10_

Antioxidants represent a significant therapeutic approach to mitigate ferroptosis and the progression of PD. CoQ_10_ is an endogenous, lipophilic cofactor distributed within cell membranes [62]. Its reduced form, ubiquinol-10 (CoQ_10_H_2_) serves as a potent scavenger of lipid peroxyl radicals and a recycler of alpha-tocopherol (Vitamin E) radicals [63]. In this context, the Ferroptosis Suppressor Protein 1 (FSP1) acts independently of GPX4 and utilizes NAD(P)H to maintain CoQ_10_ in its reduced-antioxidant state within the lipid bilayer, thereby inhibiting the propagation of lipid peroxidation [64]. Emerging clinical evidence suggests that this defense system is compromised in PD patients, as shown by the decreased serum CoQ_10_ levels and a diminished CoQ_10_H_2_/CoQ_10_ ratio [62].

Despite the clear biochemical rationale for CoQ_10_ supplementation in suppressing ferroptosis, clinical trials have largely failed to translate these mechanistic insights into therapeutic efficacy. Early pilot studies with oral administration of 200 mg CoQ_10_ for three months, or in combination with Vitamin E for one month, reported no significant improvements in UPDRS motor scores, despite achieving increased plasma CoQ_10_ concentrations [[65], [66], [67]]. These results were further substantiated by a larger clinical trial involving 600 patients, which also found no beneficial effect in CoQ_10_/vitamin E co-administration compared to placebo [68]. A later review discussing these trials questioned if CoQ_10_ reached the brain in these studies, which is an important consideration when evaluating the efficacy of these treatments [69]. Even vitamin E has limitations. Although it is a lipophilic antioxidant with some capacity to reach the brain, variable target engagement may limit its translational potential [70]. Although CoQ_10_ is a fundamental component of the cellular antioxidant network due to its synergy with Vitamin E and FSP1, current clinical trials suggest that CoQ_10_ supplementation is not sufficient to slow the progression of PD. These trials have been limited by uncertain CNS bioavailability, modest brain-target engagement, and the challenge of demonstrating disease modification once PD is established, so additional attention to the pharmacology of these treatments is vital to produce a successful therapeutic.

##### 3.2.3.2 Vitamin A (retinoic acid)

Recent research identified Vitamin A, specifically its active metabolite retinoic acid, as a specialized “gatekeeper” against ferroptosis. Retinoic acid has been shown to rescue ferroptosis-dependent growth defects in *C. elegans* after iron overload [71], while it suppresses ferroptosis induced by both RSL3-and imidazole ketone erastin (IKE), a potent derivative of erastin, in HT-1080 cell lines with efficacy comparable to ferrostatin-1 [71]. Interestingly, its anti-ferroptotic activity is not attributed to direct radical-scavenging but from activation of the Retinoic Acid Receptor (RAR), which transcriptionally upregulates essential anti-ferroptotic defense genes such as GPX4 and FSP1 [71].

In murine models of acute liver injury, retinoic acid provides antioxidative and iron-regulatory effects by reducing labile iron accumulation, inhibiting ROS and lipid peroxidation production, and increasing the expression of the antioxidative transcription factor Nrf2 and its downstream antioxidative genes HO-1 and GPX4. Furthermore, retinoic acid effectively inhibits ferroptosis in vitro following RSL3, erastin, and LPS treatment [72]. Despite these encouraging preclinical findings, epidemiological and clinical studies have yet to establish a definitive association between Vitamin A and PD risk [73]. This gap may be attributed to the brain's rapid degradation of retinoic acid by the enzyme CYP26B1, which limits the achievement of therapeutically effective concentrations [74]. Consequently, current strategies are shifting away from standard vitamin A supplementation and toward drugs that inhibit CYP26B1 to protect dopaminergic neurons more effectively.

##### 3.2.3.3 Vitamin E (α-tocopherol)

As a primary lipid-soluble antioxidant and one of the eight naturally occurring vitamin E isoforms, α-tocopherol functions as a potent inhibitor of ferroptosis. Its endogenous metabolite, α-tocopherol hydroquinone, reduces the non-heme iron within 15-lipoxygenase (15-LOX) [75], thereby limiting the generation of the toxic lipid by-product 4-HNE. In a pentylenetetrazol (PTZ)-induced rat seizure model, vitamin E inhibited 15-LOX activity and ferroptosis, resulting in decreased iron accumulation and lipid peroxidation [76]. Similarly, in a cellular model of spinal cord injury, it mitigated mitochondrial damage and downregulated ferroptosis markers like Ptgs2 [77]. Although it is clear there is a link between vitamin E and ferroptosis, there have been few clinical trials assessing its ability to slow the progression of PD. The DATATOP trial in early-stage PD patients treated with a combination of vitamin E and selegiline found no delay of motor symptoms or in needing to start levodopa therapy [78]. A more recent study combining omega-3 and vitamin E supplementation showed improved UPDRS scores and GSH levels but lacked the statistical power to confirm any disease-modifying effects [79]. Current clinical interest is focused on an ongoing 104-week Phase II clinical trial in 100 PD patients treated with tocotrienols, which are vitamin E isoforms with superior blood-brain barrier permeability (NCT04491383).

##### 3.2.3.4 Vitamin K

Vitamin K comprises a family of lipid-soluble quinones with potent anti-ferroptotic properties. Particularly, phylloquinone (vitamin K1), menaquinone (vitamin K2) and the synthetic water-soluble analog menadione have shown to protect cells from ferroptosis induced by genetic and pharmacologic inhibition of *Gpx4* or by glutamate-mediated inhibition of System Xc^−^ [80]. The antioxidant activity of vitamin K is primarily driven by its quinone head group, since dimethylmenadione, a redox-inactive form, failed to prevent ferroptosis. Mechanistically, the anti-ferroptotic activity of vitamin K dependents on FSP1, which facilitates the reduction of vitamin K to its hydroquinone form to trap lipid radicals. Consistently, genetic ablation or pharmacological inhibition of FSP1 significantly increased the concentration of vitamin K required to prevent ferroptosis [80]. Pretreatment with vitamin K2 in a murine model of acute lung injury has shown to reduce tissue iron and MDA concentrations while restoring GSH and GPX4 levels [81]. Similarly, vitamin K1 dose-dependently inhibited RSL3-and erastin-induced ferroptosis in vitro and prevented ferroptosis in an in vivo acute kidney injury model, effectively compensating for impairment of the FSP1 defense system [82]. Despite this growing preclinical evidence between vitamin K and ferroptosis, clinical translation in Parkinson's disease remains limited. To date, only one early-phase clinical trial has evaluated vitamin K2 in PD patients (PD-K2) (DRKS00019932), but no results have been published yet. Although vitamin K monotherapy is unlikely to address the multifactorial pathogenesis of PD, its ferroptosis-inhibiting properties may represent a valuable component of future multi-target therapeutic strategies.

##### 3.2.3.5 Nrf2 transcription pathway

The transcription factor nuclear factor (erythroid-derived 2)-like-2 (Nrf2) is a master regulator for >100 antioxidant, cytoprotective, and tissue-repair proteins, including HO-1, NQO-1, and GCLM [83]. Postmortem analyses of PD brains show reduced Nrf2-dependent responses [84], suggesting that a compromised Nrf2 signaling weakened cellular defenses favoring oxidative stress, α-synuclein aggregation, and consequent dopaminergic neuron loss. Beyond its general antioxidant roles, Nrf2 directly modulates ferroptotic sensitivity by binding to the SLC7A11 promoter, thereby upregulating both system Xc^−^ and GPX4 expression [85].

Several Nrf2 activators have shown promising anti-ferroptotic actions in preclinical PD models. For instance, the iridoid glycoside morroniside has shown to activate the Nrf2 pathway and prevent dopaminergic neurodegeneration in the MPTP mouse model of PD by inducing anti-ferroptotic gene expression and improving motor performance [86]. Similarly, the anti-cancer drug lapatinib has emerged as a potential disease-modifying candidate. In a rotenone rat model of PD [87], lapatinib mediated Nrf2 activation preserving GSH levels and Gpx4 mRNA expression, and suppressing the upregulation of acyl-CoA synthetase long-chain family member 4 (ACSL4), thereby limiting lipid peroxidation. Furthermore, dimethyl fumarate (DMF), a clinically approved Nrf2 activator for multiple sclerosis and psoriasis, has also demonstrated anti-ferroptotic and neuroprotective effects in an AAV6-mediated α-synuclein overexpression mouse model of PD. In this context, DMF upregulated Nrf2-dependent gene expression, prevented nigrostriatal neuronal loss and yielded modest improvements in motor performance [88]. Collectively, these findings suggest that Nrf2 activators represent a particularly promising strategy to suppress ferroptosis while simultaneously targeting other pathogenic mechanisms in PD.

#### 3.2.4 Convergence of other PD-related pathogenic mechanisms in ferroptosis

##### 3.2.4.1 LRRK2 activation

Leucine-rich repeat kinase 2 (LRRK2) is widely accepted as a protein of interest for PD. Mutations in the LRRK2 gene cause an increase in LRRK2 kinase activity and is considered both a risk variant and causes familial PD. In addition, LRRK2 kinase activity has been shown to be increased in the brains of PD patients without a LRRK2 mutation [[89], [90], [91]]. LRRK2 is recruited to stressed lysosomes, where it can phosphorylate various Rab proteins altering lysosomal positioning, secretion, and trafficking to mitigate stress [[92], [93], [94]]. Lysosomal dysfunction also releases iron from degraded ferritin, promoting ferroptosis mainly through ferritinophagy [95,96].

Sustained aberrant LRRK2 kinase activity can lead to oxidative stress, lipid peroxidation, lysosomal dysfunction and accumulation of α-synuclein, either directly or indirectly [39,97,98]. Preclinical studies suggest increased LRRK2 kinase activity may propagate ferroptosis. Specifically, the Xc^−^-GSH–GPX4 axis can mediate LRRK2-dependent microglial ferroptosis and the downstream inflammatory responses [99]. Moreover, in BV2 microglial cells, the LRRK2 inhibitor PF-06447475 decreased Fe^2+^ levels, ROS production, and 4-HNE accumulation, while increasing SLC7A11 and GPX4 expression. Conversely, overexpression of GPX4 prevented ferroptosis induced by LRRK2 co-overexpression in these cells [99].

LRRK2 can regulate microglial neuroinflammation and ferroptosis though the p62-Keap1-Nrf2 signaling pathway. While erastin increased LRRK2 expression and promoted microglial activation and ferroptosis, these effects were reversed by the ferroptosis inhibitor, Ferrostatin-1. Pharmacological inhibition of LRRK2 with PF-06447475 effectively decreased multiple drivers of ferroptosis such as Fe^2+^ accumulation, ROS and MDA levels, and restored GSH concentrations and Nrf2 signaling [100]. Consistent with these preclinical data, LRRK2 mutations carriers exhibit elevated cerebral iron levels and increased lipid peroxidation, a phenotype that could be rescued by the iron chelator deferoxamine [101]. LRRK2 inhibitors represent a promising therapeutic strategy for PD [98,102] NEU-411 is a brain-penetrant, selective LRRK2 kinase inhibitor currently under phase II clinical trials (NCT06680830), while BIIB122 (eblastin) is currently being studied in the Phase IIb LUMA study and is estimated to be completed in early 2026 (NCT05348785) [103]. While early-phase results for other inhibitors such as DNL201 (NCT03710707) and BIIB094 (NCT03976349) are still pending, LRRK2 inhibition represent a promising strategy to slow the progression of PD, though its efficacy may be constrained by the multiplicity of pathogenic mechanisms driving disease progression.

##### 3.2.4.2 NOX2 activation

NADPH oxidase 2 (NOX2) is a superoxide-generating enzyme complex [104], that plays a key role in the pathogenesis of PD [105]. Pathological activation of NOX2 in PD leads to oxidative stress, LRRK2 hyperactivation, impaired mitochondrial protein import, and formation of oxidative stress-related post-translational modifications of α-synuclein, and ultimately dopaminergic neuron loss [105]. By increasing intracellular superoxide levels, NOX2 serves as a catalyst for lipid peroxidation, heightening cellular vulnerability to ferroptosis [66,106,107]. This is supported by findings that microglial complement receptor 3 (CR3) regulates neuronal ferroptosis though NOX2-mediated iron accumulation in rotenone-treated mice [108]. While NOX2 inhibitors have yet to enter clinical trials for PD, preclinical evidences using the brain-permeable bridged tetrahydroisoquinolines as selective NADPH oxidase 2 (Nox2) inhibitors activity and decrease 4-HNE levels in a sub-acute rotenone model of PD [109,110]. These findings collectively position NOX2 as a critical upstream driver of oxidative stress and ferroptotic vulnerability in PD, highlighting this enzyme complex as a promising, albeit as yet clinically unexplored, therapeutic target.

##### 3.2.4.3 Mitochondrial dysfunction

Mitochondrial dysfunction is a hallmark of PD pathophysiology. Oligomeric α-synuclein can localize to mitochondria and interact with the TOM40/TOM20 translocase complex, thereby impairing protein import and leading to mitochondrial dysfunction and ROS production [111]. Mitochondria-derived ROS can then react with PUFAs to generate lipid peroxides that trigger ferroptosis [112]. This mechanism is exemplified by classical mitochondrial toxins such as rotenone, 1-methyl-4-phenyl-tetrahydropyridine (MPTP), and 6-hydroxydopamine (6-OHDA) which induce PD-like neurodegeneration mainly through electron transport chain disruption and subsequent ROS leakage [[113], [114], [115]].

Despite the clear link between protein import failure and neuronal death, therapeutic strategies specifically targeting the restoration of mitochondrial protein import impairment remain scarce. Instead, pharmacological interventions have largely focused on neutralizing the resulting oxidative stress via mitochondria-targeted antioxidants. For instance, MitoQ has shown some positive results in preclinical models of PD, preserving mitochondrial morphology in SH-SY5Y cells treated with 6-OHDA [116], and preventing the depletion of nigrostriatal dopamine metabolites and motor impairments in MPTP-treated mice [117]. However, these robust preclinical results have not yet effectively translated into PD patients, since in a clinical trial MitoQ failed to improve UPDRS scores despite being well-tolerated (NCT00329056) [118]. Consequently, current research is diversifying toward radical-trapping antioxidants and compounds that target broader signaling mechanisms both upstream or downstream of mitochondrial ROS production.

##### 3.2.4.4 Alpha-synuclein

α-Synuclein is a key pathogenic driver in PD that has been mechanistically connected to ferroptosis. α-Synuclein levels directly correlated with neuronal vulnerability, while its depletion protected neurons from ferroptosis, SNCA gene triplication increased their sensitivity to ferroptosis [119]. This relationship extends to neuroinflammation, where the ferroptosis inducer erastin induced microglial activation that was exacerbated by α-synuclein [100]. At the transcriptomic level, the synergy between α-synuclein overexpression and RSL3 exposure upregulates pro-ferroptotic genes such as Tfrc, Alox5, and Alox15, while downregulating anti-ferroptotic genes such as Gpx4, Slc7a11, and Pla2g6. These transcriptional changes are mirrored in the A53T mouse model of PD following treatment with the ferroptosis inducer sorafenib [33].

Beyond gene regulation, α-synuclein promotes the generation of superoxide and hydrogen peroxide, catalyzing the lipid peroxidation that defines ferroptosis [33,120]. Furthermore, α-synuclein accumulation has been shown to suppress Nrf2 activation, thereby stripping the cell of a primary antioxidant defense mechanism against ferroptosis [121].

The clinical landscape for α-synuclein-targeted therapies is rapidly evolving. Phase 2a clinical trials with risvodetinib, a c-Abl tyrosine kinase inhibitor designed to reduce α-synuclein aggregation, showed promising results by attenuating synuclein pathology in treatment-naive PD patients. Subsequent Phase 2b/3 trials scheduled for 2026 are poised to evaluate brain α-synuclein clearance more definitively [122]. In addition, the translational inhibitor of multiple neurotoxic proteins buntanetap has demonstrated positive outcomes in Phase 3 trials for early-stage PD patients by inhibiting the formation of α-synuclein oligomers and aggregates (NCT05357989) [123]. In the preclinical pipeline, α-synuclein antisense oligonucleotides (ASOs) targeting *Snca* can prevent and even reverse the progression of α-synuclein-mediated pathology in PD murine models, effectively delaying the loss of TH-positive neurons [124].

Immunotherapy represents another novel frontier for targeting α-synuclein, with various vaccines and monoclonal antibodies in clinical development. The synthetic peptide vaccine UB-312 targets insoluble α-synuclein proteins and has progressed to Phase 2 trials (NCT05634876). Other peptide-based vaccines, such as the Affitope series, elicit immune responses against oligomeric forms of α-synuclein. For instance, PD01A has shown a good safety profile and a 51% reduction in cerebrospinal fluid (CSF) α-synuclein oligomers in Phase 1 trials [125,126]. This candidate has since been optimized and renamed ACI-7104.056 for ongoing Phase 2 clinical trials (NCT06015841).

Additionally, the humanized IgG1 monoclonal antibody prasinezumab for targeting aggregated α-synuclein has moved into Phase 2b evaluations (NCT04777331) following Phase 2 data that suggested a slower decline in motor function despite negligible changes in CSF α-synuclein levels (NCT03100149) [[127], [128], [129], [130]].

Ultimately, α-synuclein inhibition remains a cornerstone of PD therapeutic strategy. Since α-synuclein is intertwined with almost every pathogenic mechanism of PD (mitochondrial import impairment, LRRK2 and NOX2 hyperactivation, and ferroptosis, among others), targeting its aggregation and accumulation provides a systemic approach to slow down the progression of a multifactorial disease as PD.

##### 3.2.4.5 Targeting multiple pathogenic mechanisms of PD

Novel therapeutic strategies for PD are increasingly focused on drug repurposing and the development of multi-target compounds capable of addressing the disease's multifaceted pathogenesis. Metformin, a staple of type 2 diabetes management, has recently emerged as a repurposed candidate for PD due to its ability to inhibit ferroptosis. Metformin attenuates mitochondrial damage and suppresses ferroptosis-associated markers such as malondialdehyde (MDA), while simultaneously upregulating the expression of the iron-exporting protein ferroportin. It further activates the Nrf2 pathway, enhances GPX4 expression, and elevates intracellular levels of glutathione (GSH) and superoxide dismutase (SOD) [[131], [132], [133]]. These biochemical shifts translate to neuroprotective outcomes in the MPTP mouse model of PD, where metformin induced neuroprotection of dopaminergic neurons and improved motor performance [134].

Ambroxol, a widely used cough suppressant, is also under investigation as repurposed therapy for PD. Mechanistically, ambroxol promotes activation of glucocerebrosidase (GCase), an enzyme that is frequently inactivated in familial PD cases carrying mutations in the GBA1 gene. A clinical trial in patients with and without GBA1 mutations demonstrated that oral ambroxol successfully crosses the blood-brain barrier to increase GCase activity within the cerebrospinal fluid and improvements in UPDRS scores. However, the absence of a placebo group for comparison limits the interpretability of these findings (NCT02941822) [135,136]. There is now a phase 3 clinical trial in progress, assessing Ambroxol to slow the progression of PD (NCT05778617).

Glucagon-like peptide-1 (GLP-1) agonists, such as liraglutide, are weight loss drugs that have shown protection against ferroptosis. In a kidney disease model, semaglutide increased GSH, while decreasing Fe^2+^, MDA, and 4-HNE. It also increased expression of anti-ferroptotic genes SLC7A11, GPX4, FSP1, FTH1, and FPN1, while decreasing pro-ferroptotic gene TFR1 in mice [137]. They have also shown promising results in PD. In a diabetic PD mouse model, liraglutide successfully inhibited necroptosis and neuroinflammation through the TNF-α signaling pathway, prevented loss of TH levels and halted the accumulation of α-synuclein, resulting in significant improvements in motor function [138]. In PD animal models, GLP-1 agonists protect dopaminergic neurons within the substantia nigra and striatum, while simultaneously mitigating oxidative stress and mitochondrial dysfunction [139]. Beyond preclinical success, these drugs have also shown promising results in clinical trials. For instance, a Phase II trial of exenatide (NCT01971242) showed improved UPDRS scores [140,141]. Similarly, clinical evaluations of liraglutide have reported both improved UPDRS and non-motor symptom scores (NCT02953665, NCT01843075), reinforcing the potential of GLP-1 agonists to address the complex pathology of PD in a clinical setting.

D-512 is a dopamine D_2/3_ receptor agonist that has shown significant neuroprotective potential in preclinical models of PD. In vitro studies using PC12 cells showed that D-512 effectively mitigated key indicators of cellular apoptosis and oxidative stress, such as nuclear condensation, DNA fragmentation, and lipid peroxidation. These findings were supported by in vivo results from an MPTP mouse model of PD, where D-512 administration successfully mitigated motor dysfunction, partially preserved dopamine levels and preserved SNpc dopaminergic neurons [142]. Although there have been no studies directly addressing the impact of D-512 on ferroptosis, the observed inhibition of lipid peroxidation suggests that D-512 may interfere with this iron-dependent cell death pathway, further establishing its potential as a multifaceted neuroprotective agent.

Small molecule nitroalkenes, such as 10-nitro-oleic acid (10-NO_2_-OA), are a promising therapeutic strategy for PD since they target multiple pathogenic events. 10-NO_2_-OA crosses the blood-brain barrier to exert neuroprotective effects in a sub-acute rotenone rat model of PD [39]. One of its primary actions is the activation of the Nrf2, which enhances the expression of cytoprotective genes such as heme oxygenase-1 (HO-1) in dopaminergic neurons. Furthermore, 10-NO_2_-OA inhibits the hyperactivation of NOX2 and LRRK2, suppressing oxidative stress and lipid peroxidation and consequent formation of toxic 4-HNE-modified α-synuclein adducts. This multi-target approach further prevents α-synuclein from binding to TOM20, thereby protecting mitochondrial protein import and maintaining cellular energy functions. Finally, 10-NO_2_-OA limits microglial activation and neuroinflammation, addressing the complex, interconnected factors that lead to nigrostriatal neurodegeneration.

A more recent study analyzed a compound, CP-50, that functions as a quinone-nitroalkene hybrid. This bifunctional molecule maintains the electrophilic action of 10-NO_2_-OA but has an additional ubiquinone group, allowing it to function as an antioxidant [143] This compound conferred protection against RSL3-indcued ferroptosis in HAECs, comparable to ferrostain-1, upregulated CYB5R1 protein expression, prevented GPX4 degradation, activated Nrf2-regulated gene expression, and suppressed NF-kB-mediated inflammation. In a mouse model of atherosclerosis, CP-50 also reduced aortic plaque area [143]. Based on these findings, CP-50 could be a promising therapeutic to prevent ferroptosis in a model of PD given the success of 10-NO_2_-OA in the rotenone model of PD. With the additional ubiquinone head group conferring protection against ferroptosis, this could provide additional protection against the pathogenic mechanisms of PD.

## 4 Conclusions

The failure of current “one-drug-one-target” therapies to provide robust clinical responses stems from the multifactorial nature of PD, where oxidative stress, mitochondrial dysfunction, α-synuclein aggregation, and neuroinflammation intersect. Because ferroptosis represents a regulated, iron-dependent cell death mechanism involved in these diverse pathogenic events, it must be prioritized in the development of next-generation disease-modifying strategies. While recent clinical trials have primarily focused on preventing ferroptosis using iron chelation to reduce metal accumulation or antioxidants to combat oxidative stress, the inherent complexity of PD suggests that these narrow interventions are insufficient to halt the loss of dopaminergic neurons in the substantia nigra pars compacta. A truly robust clinical approach requires a multi-target approach capable of neutralizing ferroptosis and simultaneously other pathogenic events to effectively halt or significantly delay the progressive neurodegeneration of PD.

## Disclosures

Dr. Straub received research funds from Bayer Pharmaceuticals and has a financial interest in Creegh and Furanica Pharmaceuticals. Dr. Fazzari has a financial interest in Creegh Pharmaceuticals.

## Funding sources

R35HL161177 (A C.S), R01HL162787 (M. F.)

## CRediT authorship contribution statement

**Lauren E. Cooke:** Writing – original draft, Writing – review & editing. **Emily Rocha:** Writing – review & editing. **Roberto DiMaio:** Writing – review & editing. **Adam C. Straub:** Writing – review & editing. **Marco Fazzari:** Writing – original draft, Writing – review & editing.

## Declaration of competing interest

Dr. Straub received research funds from Bayer Pharmaceuticals and has a financial interest in Creegh and Furanica Pharmaceuticals. Dr. Fazzari has a financial interest in Creegh Pharmaceuticals.

## Data Availability

No data was used for the research described in the article.
